# The Effects of a 4-Week, Intensified Training, and Competition Period on Salivary Hormones, Immunoglobulin A, Illness Symptoms, and Mood State in Elite Synchronised Swimmers

**DOI:** 10.3390/sports5030064

**Published:** 2017-09-01

**Authors:** Amy Tanner, Shannon Day

**Affiliations:** Department of Psychology and Sports Sciences, University of Hertfordshire, Hatfield AL10 9EU, UK; shannon@surfingcb.co.uk

**Keywords:** synchronised swimming, cortisol, testosterone, mucosal immunity, URTI

## Abstract

Given the limited research into the physiological and psychological demands of elite synchronised swimming, the aim of this study was to examine 10 elite female synchronised swimmers and analyse the relationship between training load, stress, illness episodes, and salivary biomarkers during a period of training and competition. Saliva samples were collected before (BASE), during an intensified training camp (CAMP), during an international competition period (COMP), and post competition recovery (REC) for analysis of cortisol, testosterone, and secretory immunoglobulin A (SIgA). Illness symptoms, Daily Analysis of Life Demands of Athletes (DALDA), and training load were also monitored. Training load significantly increased from BASE during CAMP and COMP (*p* < 0.01), and SIgA secretion was higher during COMP compared to BASE and CAMP (*p* < 0.01). There was no change in salivary testosterone; however, salivary cortisol was elevated during COMP compared to BASE (93%, *p* < 0.05). DALDA ‘a scores’ were correlated with salivary cortisol (r = 0.429, *p* = 0.0001). The study demonstrates that a short period of intensified training and competition did not have a detrimental effect on mucosal immunity in elite synchronised swimmers; however, swimmers displayed higher cortisol levels during the competition and increased stress symptoms.

## 1. Introduction

At an elite level, synchronised swimmers are required to undertake a high-volume and intensity of training, averaging 30–40 h per week over at least six days [1,2]. Given the high volume training demands, synchronised swimmers undergo periods of functional overreaching which are necessary to elicit adaptations and improve performance. However, there is the potential to progress to non-functional overreaching or overtraining syndrome and maladaptive outcomes [3]. Assessing the fatigue status of athletes is crucial to monitoring training adaptations and avoiding the development of non-functional overreaching, illness and injury [4].

Indicators of fatigue and recovery have been investigated including hormonal and immunological parameters [3], together with psychological questionnaires [5]. Fatigue-related mechanisms are still debated in the literature, but the importance of the hypothalamic pituitary-adrenal and gonadal axes in the regulation of metabolism and homeostasis is confirmed [3]. Testosterone (T) and cortisol (C) levels have been proposed as indicators of the balance between anabolic and catabolic processes and the physiological strain of training [6]. In elite athletes, an increase has been seen in C levels in those undertaking significant amounts of intense training in football and swimming [7,8], although other studies have shown equivocal results [9]. Chronically decreased T and increased C concentrations are suggested as indicative for a disturbance in the anabolic-catabolic balance, with repeated exercise bouts and insufficient periods of recovery shown to cause a decrease in the T/C ratio [3]; however, changes in this ratio alone should not be used as a diagnostic marker of non-functional overreaching or overtraining [10], and a multifaceted approach is required to elucidate the conditions, given the varied symptoms athletes may present.

Studies have shown that psychological stress as a result of competition may also lead to elevated C levels in performers [8,11]; however, more research is required to examine the hormonal response to competition in elite female athletes. Mood state can be a useful parameter to monitor fatigue and overreaching [12,13], and research has shown that the perception of symptoms and sources of stress are increased in the Daily Analysis of Life Demands of Athletes (DALDA) questionnaire in response to intense training [14,15]; therefore, this may be a potential tool to monitor adaptation to training and health in young athletes.

Literature also suggests that intense periods of training and competition may result in immunosuppression [16]. Engaging in long-term strenuous training has been reported to result in decreased salivary levels of secretory immunoglobulin A (SIgA) in trained athletes [17]. This may lead to an increased susceptibility to opportunistic infections, in particular upper-respiratory tract infections such as colds and influenza [17,18]. Despite studies showing a reduction in SIgA secretion in swimmers [19], another reported short-term increases in SIgA concentration during the run up to a competition [20] and, to our knowledge, there has been no research examining the effect of an intensified training and competition period in elite synchronised swimmers.

In training studies, a link has been observed between excessive training load and injury and illness occurrence, highlighting the importance of monitoring an athlete’s ability to cope with training and competition [4,21]. Given the limited research into the physiological and psychological demands of elite synchronised swimming, this research aims to present a study of an elite team of swimmers over an intensified training and competition period, with analysis of the relationship between training load, stress, illness episodes and salivary biomarkers in these athletes.

## 2. Materials and Methods

**Figure 1:**
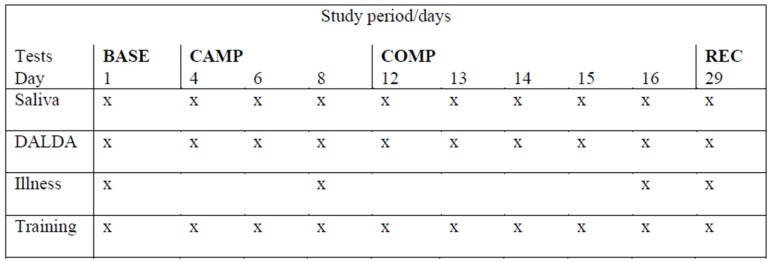
Schematic to show the study timescale and sampling points.

The experimental approach of this study was a repeated measures design, where all athletes engaged in 4-weeks of training and competition, with weekly modifications of training load. Resting morning saliva samples were collected within one hour of awakening, before a training camp (BASE) and during a training camp (3 measurements; CAMP), during an international competition (5 measurements; COMP), and two weeks post competition (REC) (Figure 1).

### 2.1. Participants

Ten female synchronised swimmers were recruited to participate in this study (age 16.8 ± 0.8 year; stature 1.66 ± 0.05 m, body mass 52.9 ± 5.8 kg, body mass index 19.2 ± 1.7 kg·m^2^). All participants were members of the Great Britain team, undergoing intense training in preparation for the European Championship qualifier. The study was approved by the University of Hertfordshire Ethics Committee and participants (and their parents or guardians if under 18 years) received written and verbal instructions and gave their written informed consent.

### 2.2. Procedures

#### 2.2.1. Training Load

Participants recorded an average daily training load during the testing period and rated each session on a rating of perceived exertion (RPE) scale of 1–10. Training load was calculated by multiplying the RPE score given by the duration of the training session in minutes [22]. For ease of analysis, average daily loads for BASE, CAMP, COMP and REC were compared.

#### 2.2.2. Saliva Collection and Analysis

Passive saliva samples were collected on 10 occasions throughout the testing period, in the morning, within 60 min of awakening. Participants were required to stop drinking 10 min before each sample collection to avoid dilution and to refrain from eating before the sample was collected. Participants provided a timed 3-min saliva sample into a pre-weighed sterile container. Samples were refrigerated at 4 °C for up to 24 h before being transported to the laboratory; samples were weighed then centrifuged at 3000 revolutions per minute for 10 min. The resulting supernatant was divided into four aliquots and stored at −80 °C prior to analysis. Saliva was analysed for SIgA, C, and T with commercially available, enzyme-linked immunosorbent assay kits (IBL, Hamburg, Germany). The sensitivity of the kits were 0.00005 µg·mL^−1^ for C, 6.1 pg·mL^−1^ for T, and 0.5 µg·mL^−1^ for IgA. The mean intra assay coefficients of variation were 5.4% for SIgA, 7.2% for C, and 11.5% for T. SIgA flow rate was determined by dividing the sample weight by sample timing, whilst secretion was calculated as concentration divided by flow rate. The samples for each player were run on the same assay to eliminate inter-assay variance. Average concentrations of salivary hormones, flow rate and SIgA concentration and secretion were recorded based on the sampling schedule (Figure 1).

#### 2.2.3. Questionnaires

At each of the 10 testing points, participants completed a DALDA questionnaire [23] and an illness episode diary. For the questionnaires, participants were asked to record their feelings at the time of completion. The DALDA was divided into parts A and B, which represent the sources of stress and the manifestation of this stress in the form of symptoms. From the DALDA questionnaires the number of ‘a’ (worse than normal) scores were reported as an average for each sampling period.

For the illness diaries, a series of URTI symptoms were listed and participants recorded if they had experienced those symptoms in the previous 7 days; and if so, whether they were light, moderate or severe. Scores were calculated by multiplying the symptom score (light = 1, moderate = 2, severe = 3) by the number of days experienced; a total score of ≥12 was classified as an illness episode [24]. Participants completed both questionnaires at the same time of each day before training or competition.

### 2.3. Statistical Analyses

Data are presented as mean and standard deviation; percentage change is also used to aid interpretation. Data were checked for normality and sphericity before statistical analysis. A one-way repeated measures analysis of variance (ANOVA) was used to examine training load, salivary measurements, and illness and DALDA scores to identify any differences over time. When the sphericity assumption in repeated-measures ANOVA was violated (Mauchly’s test), a Greenhouse-Geisser correction was used. Significant differences were assessed with Student’s paired *t*-test with Bonferoni post-hoc adjustments for multiple comparisons. Pearson’s product moment correlation coefficient was used to assess correlations between salivary C, IgA, the illness symptom score and the DALDA a scores (pooled data ±95% confidence intervals). Statistical significance was accepted at *p* < 0.05.

## 3. Results

### 3.1. Training Load

A one way ANOVA revealed a significant difference in training load between the study periods. Average training load was significantly higher during COMP (4136 ± 144) compared to BASE (1072 ± 210, 286% ± 11%, *p* < 0.001), CAMP (3092 ± 77, 34% ± 2%, *p* < 0.001), and REC (758 ± 274, 446% ± 36%, *p* < 0.001). Furthermore, training load was significantly higher during CAMP compared to BASE (188% ± 7%, *p* < 0.001) and REC (305% ± 10%, *p* < 0.001). There was no difference between BASE and REC.

### 3.2. Salivary IgA and Flow Rate

**Figure 2:**
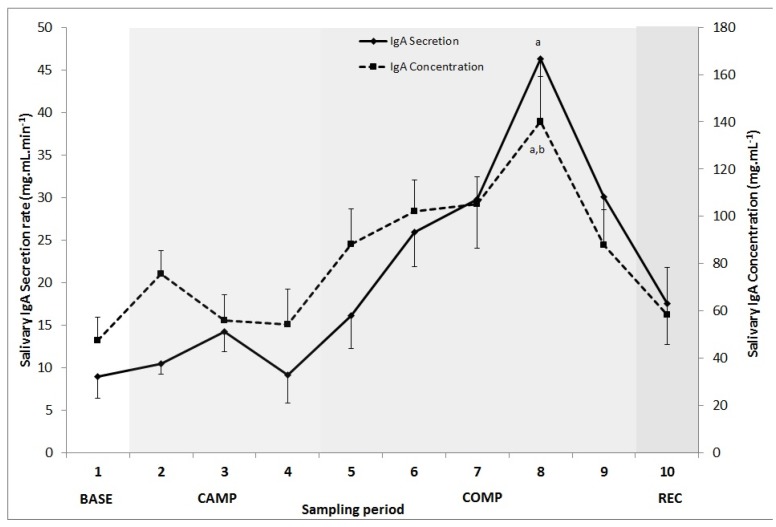
Mean ± SEM (*n* = 10) for salivary IgA secretion and concentration during baseline, training camp, competition, and post-competition testing periods; ^a^ denotes significant difference from recovery (*p* < 0.05); ^b^ denotes significant difference from camp (*p* < 0.05).

Salivary flow rate, SIgA concentration and secretion rates are presented in Table 1. A repeated measures ANOVA revealed IgA concentration was significantly higher during COMP compared to BASE (*p* = 0.012) and CAMP (*p* = 0.006), and there was a trend towards a difference between COMP and REC (*p* = 0.086). Furthermore, within COMP (samples 5–9) there was a significant increase from sample 5 to point 8 on the 4th day of the COMP and also from sample 6 to 8 (Figure 2). There was no significant difference between any other time points.

Salivary SIgA secretion rate was significantly higher during COMP compared to BASE (243% ± 328%, *p* < 0.001) and CAMP (173% ± 145%, *p* = 0.008); however, secretion decreased from COMP to REC (43% ± 56%, *p* > 0.05). Within COMP, SIgA concentration significantly increased from day 1 to 4 (samples 5 to 8, Figure 2).

Saliva flow rate was compared across time points and there was a trend towards a higher flow rate during COMP and REC compared to BASE (*p* = 0.071 and *p* = 0.072 respectively). Flow rate was 58% ± 52% higher during COMP and 84% ± 78% higher during REC compared to BASE. Within COMP there was a trend for saliva flow rate to be higher on day four (0.36 ± 0.14 mL·min^−1^) compared to day two (0.28 ± 0.13 mL·min^−1^, *p* = 0.077). BASE and CAMP measurements were not significantly different.

### 3.3. Salivary C and T

Salivary C and T response is presented in Table 1. A repeated measure ANOVA revealed a trend towards a significant difference between time points. Further analysis showed that C was significantly higher during COMP compared to BASE (93% ± 64%, *p* = 0.04) and there was a significant decrease following REC (47% ± 37%, *p* = 0.03). There was also a significant increase from CAMP to COMP (97% ± 103%, *p* = 0.002). There was no difference between any other time points. There was a significant correlation between the salivary C and SIgA secretion rate (r = 0.41, 0.22–0.57, *p* < 0.001) and concentration (r = 0.48, 0.31–0.63, *p* < 0.001). When examining salivary T, there was no significant difference between any time points.

### 3.4. DALDA

**Figure 3:**
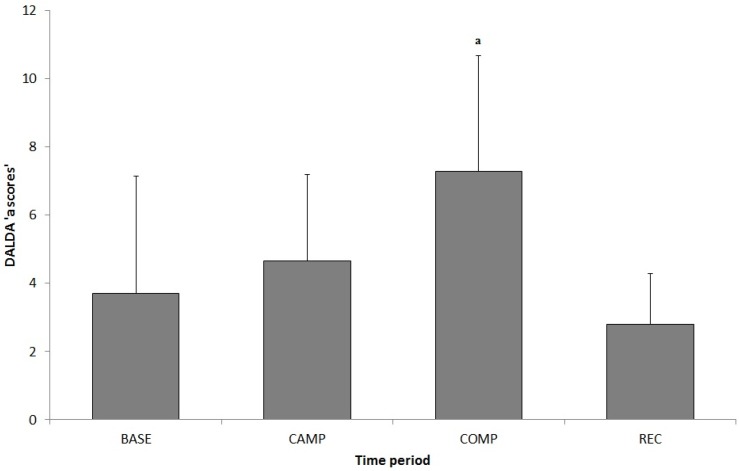
Mean ± SD (*n* = 10) for Daily Analysis of Life Demands of Athletes (DALDA) ‘a scores’ during BASE, CAMP, COMP, and REC testing periods; ^a^ denotes significant difference from REC (*p* < 0.05).

There was a significant difference between training periods and DALDA ‘a scores’ (worse than normal) (Figure 3). During COMP there was a trend towards more ‘a scores’ than BASE (*p* = 0.069) and significantly more ‘a scores’ than REC (*p* = 0.01). There was no significant difference between any other time points. Furthermore, there was a significant moderate correlation between DALDA ‘a scores’ and cortisol concentration (r = 0.43, 0.24–0.58, *p* < 0.001).

### 3.5. Illness Episodes

During the study period there were a total of three illness episodes in different athletes, two were experienced during BASE and one during COMP. There was no difference in symptom score between any time points (Table 1). Furthermore, there was no correlation between symptom scores and IgA concentration, secretion rate or DALDA score.

## 4. Discussion

The aims of this study were to investigate the salivary immune and endocrine response to an intensified training CAMP and international COMP in elite synchronised swimmers and to examine psychological stress, illness, and training load during this period. To the best of the research team’s knowledge, this is the first study to examine these parameters in synchronised swimmers during a training and COMP period.

During the study, there were a greater number of ‘worse than normal’ responses reported in the DALDA questionnaires during COMP and this reduced after REC. This represented an increase in stress symptoms experienced during the COMP period. There was no change during CAMP, which suggested the athletes were able to cope with the five days of increased training; these results were similar to those observed in futsal players [25]. The number of ‘worse than normal’ DALDA scores was also correlated with salivary C during the study period; this is supported by other research reporting a correlation between C and stress symptoms [26,27].

Salivary C was only elevated during COMP. Identified as a marker of hypothalamus-pituitary-adrenal axis activity, C has been shown to be moderately correlated with perceived stress [28]. Furthermore, the results are in agreement with many studies that have reported an increase in C in response to competition stress [11,29]. Despite the significant increase in training load during CAMP, there was no concomitant change in C, suggesting the elevation during COMP was likely to be caused by psychological and situational factors rather than physical exertion. The young athletes were in a foreign unfamiliar environment, participating in an international competition with more pressure to achieve optimal outcomes. In addition, during COMP they had limited contact with their families and these factors would have undoubtedly caused psychological stress.

Dissimilarly, salivary T did not change during the testing period. This is in contrast to research that reported elevated T in response to intensive training and competition in rugby [30]. The response of T prior to a competition is not well established in the literature in regards to female athletes. However, in agreement with current findings, no change in T was found before other competitive situations [31]. Although, it is important to note that testosterone is found in low levels in biological fluids, particularly saliva, and this make it difficult to quantify extremely low levels found in some women, potentially invalidating the results [32].

It has been proposed that the use of free C and T levels gives an indication of the anabolic/catabolic balance in response to training [33]. There was no change in C in some athletes, emphasising the importance of monitoring individuals to establish resting baseline values for comparison over the season. Given the short timescale of this study, individual analysis was not conducted; however, future research should endeavor to monitor athletes individually to look for trends in physiological and psychological responses to training and competition. Additionally, some athletes may have coped with the training load and competition period better than others and there is evidence athletes can become accustomed to training stress [34]. In women, it is also important to consider the menstrual cycle and oral contraceptive use in hormone values, particularly T, although most of the variations in anabolic/catabolic balance have been reported as a result of C changes [35]. Given speculation that oral contraceptive use may impact the free C response to physical and psychological stress, and in light of evidence that mucosal immunity may be affected by these stresses, this area requires further research [36,37]. Furthermore, a lack of information about the athlete’s menstrual cycles is a limitation to this study and future research should control for this parameter.

During the present study salivary IgA concentration and secretion increased during and throughout COMP. Many studies have found a decrease in SIgA in response to competition and intense training [16,19], and this has been linked with an increased susceptibility for infection; however, the present study does not support those results. Some studies have observed an acute increase in response to exercise [38], and there is speculation that this increase might be attributed to β-sympathetic actions on the salivary glands [39]; therefore, the increase in the present study could perhaps represent an increased sympathetic nervous system activation in response to COMP. In an attempt to explain the findings, salivary flow rate was examined, and there was a small increase from BASE to COMP; however, with an increased flow rate you would expect a decrease in SIgA secretion due to a dilution effect [38], but there was an increase in the secretion during this period due to a rise in absolute concentration. An alternative explanation could be linked to the psychological strain on the athletes as a result of the competition. Some studies have reported an increase in SIgA in response to acute psychological stressors [40]. Given the increase in stress symptoms reported in the DALDA, the athletes were assessed to be under a large amount of psychological strain and therefore the increase in SIgA may represent an acute increase in response to stressors experienced during and before the competition. In future studies it would be useful to employ some other tools to examine the psychological mood state, such as interviews, in order to confirm these findings and gain more knowledge about the athletes’ emotions and coping mechanisms.

In addition, some studies have hypothesised that C may influence the secretion of IgA; however, mechanisms for this are yet to be established. In the present study there was a moderate correlation between both SIgA secretion and concentration and C, but this is in contrast to previous studies that found no relationship [41,42], and further examination of the correlation and individual trends are required in future studies. In the present study, there was no link between illness and mucosal immunity, but the small sample size may have affected statistical power, as participant numbers were lower than previous immune studies [16] but in line with hormone studies [8]. Furthermore, the relatively short duration of the study could explain this finding, especially as most are conducted over months with more opportunity to record illness. Monitoring training load with session RPE is also not without limitations; some studies have shown that the session RPE is a valid and reliable measure of training load during constant load exercise [43]; however, it can be affected by the timing of completion [44]. Ideally, heart rate would have been noted for each session, but this was not feasible in the current study.

In conclusion, results suggest that in elite synchronised swimmers, stress symptoms can be monitored during a competition period and these may relate to training load and physiological markers such as cortisol and IgA. Moreover, a short period of intense training and competition does not appear to have a detrimental effect on mucosal immunity in these swimmers. Further longer term monitoring is advised to examine mucosal immunity and knowledge of the menstrual cycle and oral contraceptive use would add more value to research conducted on female athletes.

## Figures and Tables

**Table 1:** Mean ± SD (*n* = 10) salivary IgA concentration, secretion rate, flow rate, and cortisol and testosterone concentration at BASE, CAMP, COMP, and REC testing periods.

Time Point (Mean ± SD)	BASE	CAMP	COMP	REC
IgA concentration (mg·mL^−1^)	47.6 ± 31.0 ^a^	62.0 ± 32.6 ^a^	107.4 ± 39.4	58.4 ± 62.8
IgA secretion rate (mg·mL·min^−1^)	9.0 ± 8.2 ^a^	11.3 ± 4.5 ^a^	30.9 ± 13.9	17.6 ±15.2
Saliva flow rate (mL·min^−1^)	0.19 ± 0.10	0.21 ± 0.07	0.30 ± 0.11	0.35 ± 0.14
Salivary C (µg·mL^−1^)	0.63 ± 0.27 ^a^	0.62 ± 0.19 ^a^	1.21 ± 0.32	0.64 ± 0.32 ^a^
Salivary T (pg·mL^−1^)	78.9 ± 37.7	68.7 ± 31.4	70.0 ± 36.4	63.5 ±39.7
Illness symptom score	7 ± 9	1 ± 1	4 ± 7	1 ± 2

^a^ significantly lower than COMP (*p* < 0.05).

## References

[1] Weinberg S.K. (1986). Medical aspects of synchronized swimming. Clin. Sports Med..

[2] Mountjoy M. (2009). Injuries and medical issues in synchronized olympic sports. Curr. Sports Med. Rep..

[3] Meeusen R., Duclos M., Foster C., Fry A., Gleeson M., Nieman D., Raglin J., Rietjens G., Steinacker J., Urhausen A. (2013). Prevention, diagnosis, and treatment of the overtraining syndrome: Joint consensus statement of the european college of sport science and the american college of sports medicine. Med. Sci. Sports Exerc..

[4] Halson S.L. (2014). Monitoring training load to understand fatigue in athletes. Sports Med..

[5] Kellmann M. (2010). Preventing overtraining in athletes in high-intensity sports and stress/recovery monitoring. Scand. J. Med. Sci. Sports.

[6] Urhausen A., Gabriel H., Kindermann W. (1995). Blood hormones as markers of training stress and overtraining. Sports Med..

[7] Minetto M.A., Lanfranco F., Tibaudi A., Baldi M., Termine A., Ghigo E. (2008). Changes in awakening cortisol response and midnight salivary cortisol are sensitive markers of strenuous training-induced fatigue. J. Endocrinol. Investig..

[8] O’Connor P.J., Morgan W.P., Raglin J.S., Barksdale C.M., Kalin N.H. (1989). Mood state and salivary cortisol levels following overtraining in female swimmers. Psychoneuroendocrinology.

[9] Passelergue P., Lac G. (1999). Saliva cortisol, testosterone and t/c ratio variations during a wrestling competition and during the post-competitive recovery period. Int. J. Sports Med..

[10] Duclos M. (2008). A critical assessment of hormonal methods used in monitoring training status in athletes. Int. Sportmed. J..

[11] Aubets J., Segura J. (1995). Salivary cortisol as a marker of competition related stress. Sci. Sports.

[12] Raglin J., Sawamura S., Alexiou S., Hassmen P., Kentta G. (2000). Training practices and staleness in 13–18-year-old swimmers: A cross-cultural study. Pediatr. Exerc. Sci..

[13] Kentta G., Hassmen P., Raglin J.S. (2001). Training practices and overtraining syndrome in swedish age-group athletes. Int. J. Sports Med..

[14] Halson S.L., Bridge M.W., Meeusen R., Busschaert B., Gleeson M., Jones D.A., Jeukendrup A.E. (2002). Time course of performance changes and fatigue markers during intensified training in trained cyclists. J. Appl. Physiol..

[15] Milanez V.F., Ramos S.P., Okuno N.M., Boullosa D.A., Nakamura F.Y. (2014). Evidence of a non-linear dose-response relationship between training load and stress markers in elite female futsal players. J. Sports Sci. Med..

[16] Gleeson M., McDonald W.A., Cripps A.W., Pyne D.B., Clancy R.L., Fricker P.A. (1995). The effect on immunity of long-term intensive training in elite swimmers. Clin. Exp. Immunol..

[17] Gleeson M., Pyne D.B. (2000). Special feature for the olympics: Effects of exercise on the immune system: Exercise effects on mucosal immunity. Immunol. Cell Biol..

[18] Engebretsen L., Soligard T., Steffen K., Alonso J.M., Aubry M., Budgett R., Dvorak J., Jegathesan M., Meeuwisse W.H., Mountjoy M. (2013). Sports injuries and illnesses during the london summer olympic games 2012. Br. J. Sports Med..

[19] Gleeson M., McDonald W.A., Pyne D.B., Cripps A.W., Francis J.L., Fricker P.A., Clancy R.L. (1999). Salivary iga levels and infection risk in elite swimmers. Med. Sci. Sports Exerc..

[20] Gleeson M., McDonald W., Pyne D., Clancy R., Cripps A., Francis J., Fricker P. (2000). Immune status and respiratory illness for elite swimmers during a 12-week training cycle. Int. J. Sports Med..

[21] Foster C. (1998). Monitoring training in athletes with reference to overtraining syndrome. Med. Sci. Sports Exerc..

[22] Foster C., Daines E., Hector L., Snyder A.C., Welsh R. (1996). Athletic performance in relation to training load. Wis. Med. J..

[23] Rushall B.S. (1990). A tool for measuring stress tolerance in elite athletes. J. Appl. Sport Psychol..

[24] Gleeson M., Bishop N., Oliveira M., McCauley T., Tauler P., Muhamad A.S. (2012). Respiratory infection risk in athletes: Association with antigen-stimulated il-10 production and salivary iga secretion. Scand. J. Med. Sci. Sports.

[25] Moreira A., de Moura N.R., Coutts A., Costa E.C., Kempton T., Aoki M.S. (2013). Monitoring internal training load and mucosal immune responses in futsal athletes. J. Strength Cond. Res..

[26] Moreira A., Arsati F., de Oliveira Lima-Arsati Y.B., Simões A.C., de Araújo V.C. (2011). Monitoring stress tolerance and occurrences of upper respiratory illness in basketball players by means of psychometric tools and salivary biomarkers. Stress Health.

[27] Gomes R.V., Moreira A., Lodo L., Nosaka K., Coutts A.J., Aoki M.S. (2013). Monitoring training loads, stress, immune-endocrine responses and performance in tennis players. Biol. Sport.

[28] Hellhammer D.H., Wüst S., Kudielka B.M. (2009). Salivary cortisol as a biomarker in stress research. Psychoneuroendocrinology.

[29] Salvador A., Suay F., Gonzalez-Bono E., Serrano M. (2003). Anticipatory cortisol, testosterone and psychological responses to judo competition in young men. Psychoneuroendocrinology.

[30] Gleeson M., Allgrove J., Reddin D. Salivary Cortisol, Testosterone and Immunoglobulin a Changes during 3 Consecutive Weeks of Training and International Competition in Elite Rugby Union Players. Proceedings of the 12th Annual Congress of the European College of Sport Science.

[31] Filaire E., Sagnol M., Ferrand C., Maso F., Lac G. (2001). Psychophysiological stress in judo athletes during competitions. J. Sports Med. Phys. Fit..

[32] Kushnir M.M., Rockwood A.L., Roberts W.L., Pattison E.G., Bunker A.M., Fitzgerald R.L., Meikle A.W. (2006). Performance characteristics of a novel tandem mass spectrometry assay for serum testosterone. Clin. Chem..

[33] Adlercreutz H., Harkonen M., Kuoppasalmi K., Naveri H., Huhtaniemi I., Tikkanen H., Remes K., Dessypris A., Karvonen J. (1986). Effect of training on plasma anabolic and catabolic steriod hormones and their response to physical exercise. Int. J. Sports Med..

[34] Vuorimaa T., Ahotupa M., Hakkinen K., Vasankari T. (2008). Different hormonal response to continuous and intermittent exercise in middle-distance and marathon runners. Scand. J. Med. Sci. Sports.

[35] Vervoorn C., Quist A.M., Vermulst L.J., Erich W.B., de Vries W.R., Thijssen J.H. (1991). The behaviour of the plasma free testosterone/cortisol ratio during a season of elite rowing training. Int. J. Sports Med..

[36] Kirschbaum C., Kudielka B.M., Gaab J., Schommer N.C., Hellhammer D.H. (1999). Impact of gender, menstrual cycle phase, and oral contraceptives on the activity of the hypothalamus-pituitary-adrenal axis. Psychosom. Med..

[37] Hayashida H., Dolan N.J., Hounsome C., Alajmi N., Bishop N.C. (2015). Salivary siga responses to acute moderate-vigorous exercise in monophasic oral contraceptive users. Appl. Physiol. Nutr. Metab..

[38] Papacosta E., Nassis G.P. (2011). Saliva as a tool for monitoring steroid, peptide and immune markers in sport and exercise science. J. Sci. Med. Sport.

[39] Chicharro J.L., Lucia A., Perez M., Vaquero A.F., Urena R. (1998). Saliva composition and exercise. Sports Med..

[40] Ring C., Carroll D., Hoving J., Ormerod J., Harrison L.K., Drayson M. (2005). Effects of competition, exercise, and mental stress on secretory immunity. J. Sport Sci..

[41] Dimitriou L., Sharp N.C.C., Doherty M. (2002). Circadian effects on the acute responses of salivary cortisol and iga in well trained swimmers. Br. J. Sports Med..

[42] Farzanaki P., Azarbayjani M., Rasaee M., Jourkesh M., Ostojic S., Stannard S., Shabestar Branch I., Jourkesh M. (2008). Salivary immunoglobulin a and cortisol response to training in young elite female gymnasts. Braz. J. Microbiol..

[43] Herman L., Foster C., Maher M., Mikat R., Porcari J. (2006). Validity and reliability of the session rpe method for monitoring exercise training intensity. S. Afr. J. Sports Med..

[44] Borresen J., Lambert M.I. (2009). The quantification of training load, the training response and the effect on performance. Sports Med..

